# Antithrombotic and antiplatelet activities of small-molecule alkaloids from *Scolopendra subspinipes mutilans*

**DOI:** 10.1038/srep21956

**Published:** 2016-02-24

**Authors:** Wonhwa Lee, JungIn Lee, Roshan Kulkarni, Mi-Ae Kim, Jae Sam Hwang, MinKyun Na, Jong-Sup Bae

**Affiliations:** 1 College of Pharmacy, CMRI, Research Institute of Pharmaceutical Sciences, BK21 Plus KNU Multi-Omics based Creative Drug Research Team Kyungpook National University, Daegu 41566, Republic of Korea; 2 BK21 Plus KNU Biomedical Convergence Program, Department of Biochemistry and Cell Biology, School of Medicine, Kyungpook National University, Daegu 41566, Republic of Korea; 3 College of Pharmacy, Chungnam National University, Daejeon, 34134, Republic of Korea; 4 Department of Agricultural Biology, The National Academy of Agricultural Science, RDA, 166 Nongsaengmyoungro, Wanju-gun, 55365, Republic of Korea

## Abstract

The aim of this study was to discover small-molecule anticoagulants from *Scolopendra subspinipes mutilans* (SSM). A new acylated polyamine (**1**) and a new sulfated quinoline alkaloid (**2**) were isolated from SSM. Treatment with the new alkaloids **1**, **2**, and indole acetic acid **4** prolonged the activated partial thromboplastin time and prothrombin time and inhibited the activity and production of thrombin and activated factor X. Furthermore, compounds **1**, **2**, and **4** inhibited thrombin-catalyzed fibrin polymerization and platelet aggregation. In accordance with these potential *in vitro* antiplatelet activities, compounds **1**, **2**, and **4** showed enhanced antithrombotic effects in an *in vivo* pulmonary embolism and arterial thrombosis model. Compounds **1**, **2**, and **4** also elicited anticoagulant effects in mice. Collectively, this study may serve as the groundwork for commercializing SSM or compounds **1**, **2**, and **4** as functional food components for the prevention and treatment of pathogenic conditions and serve as new scaffolds for the development of anticoagulants.

Cardiovascular diseases and thromobosis are the leading causes of death worldwide^1^. Thrombus-induced myocardial infarction or ischemic stroke is the main cause of cardiovascular diseases (CVD)-related death. Thrombus formation is a crucial event in the pathophysiology of atherosclerotic cardiovascular diseases^1^. Thrombus formation due to an abnormal coagulation process is often observed in arteries or veins and may result in reduced blood flow or ischemia^1^. Platelet activation in atherosclerotic arteries is central to the development of arterial thrombosis; therefore, a precise control of platelet function is imperative in preventing thrombotic events^2^. The insufficient antithrombus and antiplatelet effect of the present armamentarium might explain the vascular relapses. Most thromboembolic processes require anticoagulant therapy. This explains the current efforts to develop specific and potent anticoagulant and antithrombotic agents. Research on novel bioactive compounds and drugs with different mechanisms of action, increased efficacy, and low toxicity is highly needed^1^.

The centipede *Scolopendra subspinipes mutilans* L. Koch (SSM) is a medicinal resource listed in the Korean Herbal Pharmacopeia and Chinese Pharmacopeia and has been used to treat stroke and stroke-related hemiplegia, epileptic seizures, tetanus, and pain^3^,^4^. The traditional application of SSM in stroke and stroke-related hemiplegia has attracted attention to discover anticoagulant agents from the centipede. Thus far, peptides and proteins in the venom of SSM have been demonstrated to have an antithrombotic effect^5^,^6^,^7^,^8^. In addition, evidence-based research on SSM resulted in the discovery of a peptide with a potential to be developed into an analgesic as effective as morphine^9^. There are only a few chemical studies on the secondary metabolites from SSM, where the quinoline alkaloids are characterized as representative small-molecule metabolites from SSM^10^,^11^,^12^,^13^.

Our study aimed to discover small-molecule anticoagulants from the whole material of SSM that has been clinically used. In this study, we examined the anticoagulant activity of the isolated compounds by evaluating the production of activated factor X (FXa) and thrombin. Furthermore, we assessed their effects on prothrombin time (PT), activated partial thromboplastin time (aPTT), and fibrinolytic activity.

## Results

### Isolation and structure determination of small-molecule alkaloids from SSM

**Figure 1:**
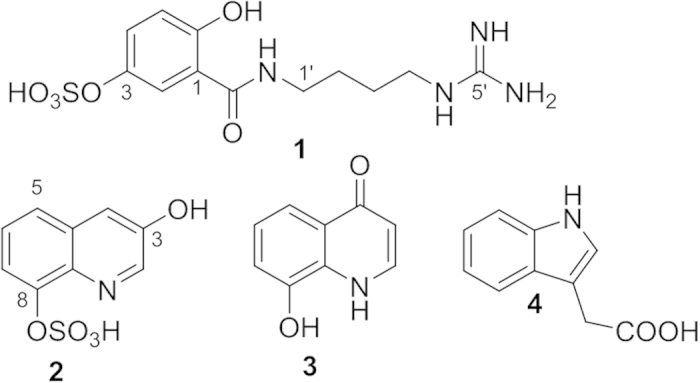
Compounds isolated from Scolopendra subspinipes mutilans.

Spectroscopic data analysis-guided isolation of the EtOH extract of SSM resulted in the purification of a series of alkaloids including two new compounds (Fig. 1). The structures of the isolated compounds were determined by MS, 1D, and 2D NMR analysis.

**Figure 2:**
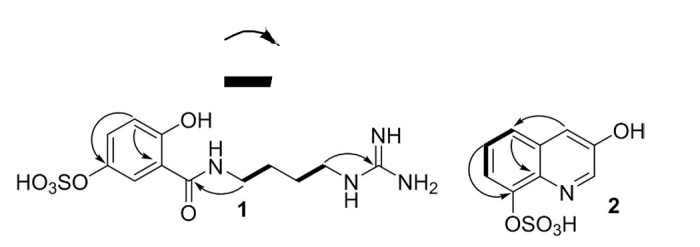
Key HMBC (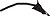) and COSY (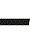) correlations of compounds 1 and 2.

Compound **1** was isolated as a yellow amorphous powder. The HR-ESI-MS data with a pseudomolecular ion peak at *m/z* 369.0841 (calculated [M+Na]^+^, *m/z* 369.0845) showed the molecular formula to be C_12_H_18_N_4_O_6_S. The ^1^H and ^13^C NMR spectra showed distinct resonances for an agmatine moiety (Figs S1, S2, S9 and S10). The NMR spectra were closely matched to those of gentisic acid (GA, Figs S1, S2, S7 and S8). Compared with the proton chemical shifts of gentisic acid, the protons of H-2 and H-4 in compound **1** significantly shifted downfield (*δ*_1_- *δ*_GA_ 0.35 and 0.31 respectively) indicating the presence of a sulfate functionality at C-3 in **1**. A strong heteronuclear multiple bond correlation (HMBC; Figs 2 and S4, S5) from the olefinic methine proton at *δ*_H_ 6.85 (H-5) to the quaternary carbon at *δ*_C_ 145.7, and the upfield carbon chemical shift about 5 ppm^14^,^15^ further supported the substitution of the sulfated group at C-3. The HMBC between H_2_-1′ at *δ*_H_ 3.44 and the carbonyl carbon at *δ*_C_ 170.8 (C-7) allowed the linkage of the agmatine and gentisic acid fragments (Figs 2 and S4), and led to the identification of **1** as gentisic acid agmatine amide-3-sulfate. Compound **1** can be classified as an acylated polyamine, a class reported in the venoms of arachnids^16^. Except for the polyamine chain, the structure of **1** is related to a toxin recently isolated from the venom of spider *Tegenaria agrestis*^17^. Comparing the structures of known acylated polyamines, compound **1** was found to be a unique chemotype.

Compound **2** was isolated as a yellow amorphous powder and showed characteristic NMR signals for jineol^18^. The molecular formula C_9_H_7_NO_5_S (calcd [M+Na]^+^, *m/z* 263.9943) determined from the pseudomolecular ion peak at *m/z* 263.9935 in the HR-ESI-MS data suggested that compound **2** is a sulfated analog of jineol. The carbon chemical shift at *δ*_C_ 149.0 (C-8), shifted upfield around 5 ppm relative to jineol, indicating the presence of a sulfate group in compound **2**. The HMBC between the proton at *δ*_H_ 7.50 (H-4) and the carbon at *δ*_C_ 123.9 (C-5) and between the proton at *δ*_H_ 7.45 (H-6) and the carbon at *δ*_C_ 149.0 (C-8) confirmed the location of the sulfate group at C-8 (Figs 2 and S14). The ^1^H-^1^H COSY correlations of H-5/H-6 and H-6/H-7 (Figs 2 and S15) supported the substitution of sulfate group at C-8. Thus, compound **2** was identified as 3-hydroxyquinolin-8-yl hydrogen sulfate, named jineol-8-sulfate.

Compound **3** was isolated as a yellow amorphous powder. In the ^1^H and ^13^C NMR spectra (Table S2), the carbonyl carbon at *δ*_C_ 181.0 along with olefinic methine groups at *δ*_H_ 6.35 (d, *J* = 7.1 Hz; *δ*_C_ 109.7) and *δ*_H_ 7.93 (d, *J* = 7.1 Hz; *δ*_C_ 140.6) indicated **3** to be a quinolin-4-one type alkaloid. The proton signals at *δ*_H_ 7.10 (d, *J* = 8.0 Hz), 7.24 (t, *J* = 8.0 Hz), and *δ*_H_ 7.70 (d, *J* = 8.0 Hz) along with an olefinic quaternary carbon at *δ*_C_ 148.5 indicated that compound **3** is a quinolone with a hydroxyl group at C-5 or 8. Based on the analysis of HMBC data (Fig. S18) showing a cross peak between the proton at *δ*_H_ 7.70 and the carbonyl carbon at *δ*_C_ 181.0, compound **3** was determined as 8-hydroxy-4-quinolone. The compound has been reported in the ink of an Octopus species *Octopus dofleini martini*^19^.

Compound **4** was isolated as a colorless solid. The NMR spectra were typical of indole-type compounds and a methylene group at *δ*_H_ 3.96 (s; *δ*_C_ 33.5) and a carbonyl carbon at *δ*_C_ 176.5 identified **4** as indole acetic acid^20^ and the structure was additionally confirmed by analysis of the HMBC spectrum (Fig. S21).

The other known alkaloids were identified as jineol^18^, 3-methoxy-8-hydroxy-quinolin-2-one^21^, 3,4-dimethoxy-8-hydroxy-quinolin-2-one^22^, and scolopendrine^23^ by comparison of their spectroscopic data with those reported in the literature.

### Effect of the isolated compounds on clotting and bleeding time

Incubation of human plasma with compounds **1**-**4** affected coagulation. The anticoagulant activity of each compound was evaluated using aPTT and PT assays and human plasma (Table 1). Although the anticoagulant activity of compounds **1**, **2**, and **4**, but not compound **3,** was weaker than that of heparin, the aPTT and PT were significantly prolonged by compounds **1**, **2**, and **4** at 1–5 μM. However, other compounds, such as jineol, 3-methoxy-8-hydroxy-quinolin-2-one, 3,4-dimethoxy-8-hydroxy-quinolin-2-one, and scolopendrine, did not alter the *in vitro* coagulation time (Table S3). Compounds **1**, **2**, and **4** at 3.30, 3.82, and 3.53 μM, respectively, doubled the clotting time in the aPTT assay and at concentrations of 3.70, 4.21, and 3.76 μM, respectively, doubled the clotting time in the PT assay. Therefore, our results indicate that compounds **1**, **2**, and **4** can inhibit the blood coagulation pathway.

To confirm these *in vitro* results, the *in vivo* tail bleeding times were determined. The average circulating blood volume for mice is 72 mL/kg^24^. Because the average weight of the mouse used in this study was 27 g and the average blood volume is 2 mL, the amount of compound **1** (1.73 or 3.46 μg per mouse), **2** (1.21 or 2.41 μg per mouse), **3** (0.81 or 1.61 μg per mouse), and **4** (0.87 or 1.75 μg per mouse) equaled a peripheral blood concentration of approximately 2.5 or 5.0 μM, respectively. As shown in Table 1, the tail bleeding times were significantly prolonged by compounds **1**, **2**, and **4** (i.v. injection) in comparison to those of the controls. The anticoagulation effect of each compound was observed *ex vivo* in mice as demonstrated by the dose-dependent prolongation of the aPTT and PT (Table 2). However, the *ex vivo* coagulation time was not altered by other compounds, such as jineol, 3-methoxy-8-hydroxy-quinolin-2-one, 3,4-dimethoxy-8-hydroxy-quinolin-2-one, and scolopendrine (Table S4).

### Effect of the isolated compounds on fibrin polymerization, platelet aggregation, and cellular viability

**Figure 3:**
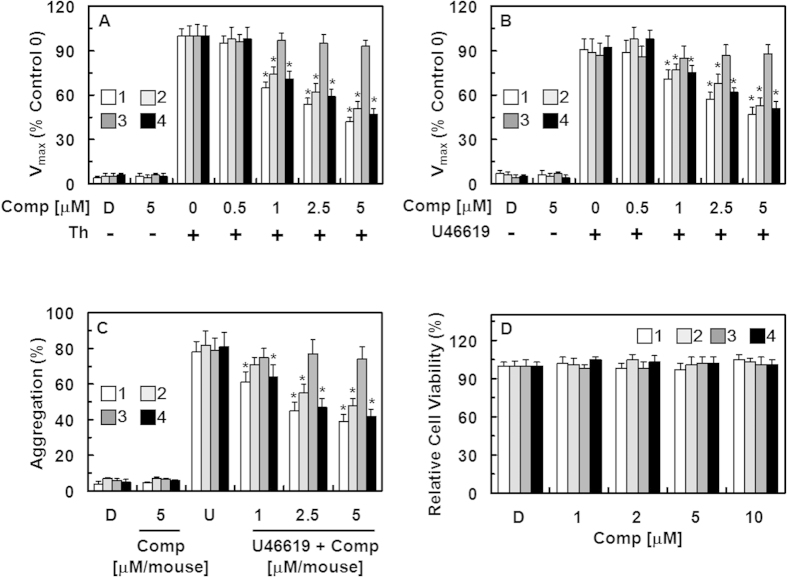
Effects of compounds 1–4 on fibrin polymerization and platelet aggregation and their cytotoxicity. (**A**) Thrombin (Th)-catalyzed fibrin polymerization was monitored at the indicated concentrations of compound **1** (white box), **2** (light gray box), **3** (dark gray box), and **4** (black box) using a catalytic assay, as described in the “Materials and Methods” section. The results are expressed as the V_max_ values as percentage of the controls. **(B**) The effect of each compound on human platelet aggregation induced by 2 mM U46619. (**C**) Each compound in DMSO was injected intravenously at the indicated concentration. The effects of each compound on mouse platelet aggregation induced by 2 μM U46619 (U) were monitored *ex vivo*. (**D**) The effect of each compound on cellular viability was measured using the MTT assay. D = 0.2% DMSO used as the vehicle control. The data represent the means ± SEM of three independent experiments performed in triplicate. ^*^p < 0.05 vs. Th (A) or U46619 (**B,C**) alone.

The effect of each compound on thrombin-catalyzed fibrin polymerization in human plasma was determined according to the changes in the absorbance at 360 nm as described in the Materials and Methods section. The incubation of human plasma with compounds **1**, **2**, and **4** resulted in a significant decrease in the maximal rate of fibrin polymerization (Fig. 3A). To eliminate the effect of the samples’ pH, all dilutions were made in 50 mM TBS (pH 7.4). We also evaluated the effect of the same volume of DMSO on human plasma and found that this did not affect coagulation. Compound **3** did not suppress thrombin-induced fibrin polymerization (Fig. 3A). The reptilase-catalyzed polymerization assay was performed to assess if the observed decrease in polymerization could be due to a direct effect on thrombin, leading to a decrease in fibrin production rather than polymerization of the formed fibrin. Compounds **1**, **2**, and **4** significantly decreased the reptilase-catalyzed polymerization (Fig. S32), excluding the potential direct effect on thrombin. To confirm the anticoagulant activities of compounds **1**, **2**, and **4**, a U46619 (a stable thromboxane A2 analog/aggregation agonist)-catalyzed platelet aggregation assay was performed. As shown in Fig. 3B, treatment with compounds **1**, **2**, and **4** significantly inhibited human platelet aggregation induced by U46619 (final concentration: 2 μM) in a concentration-dependent manner. These *in vitro* results were confirmed in an *ex vivo* platelet aggregation assay (i.v. injection, Fig. 3C). Furthermore, no compound affected cell viability at concentrations up to 10 μM (Fig. 3D) as determined using the MTT assay in HUVECs treated with the compounds for 24 h.

### *In vivo* effects of the isolated compounds in an arterial thrombosis and a pulmonary thrombosis model

**Figure 4:**
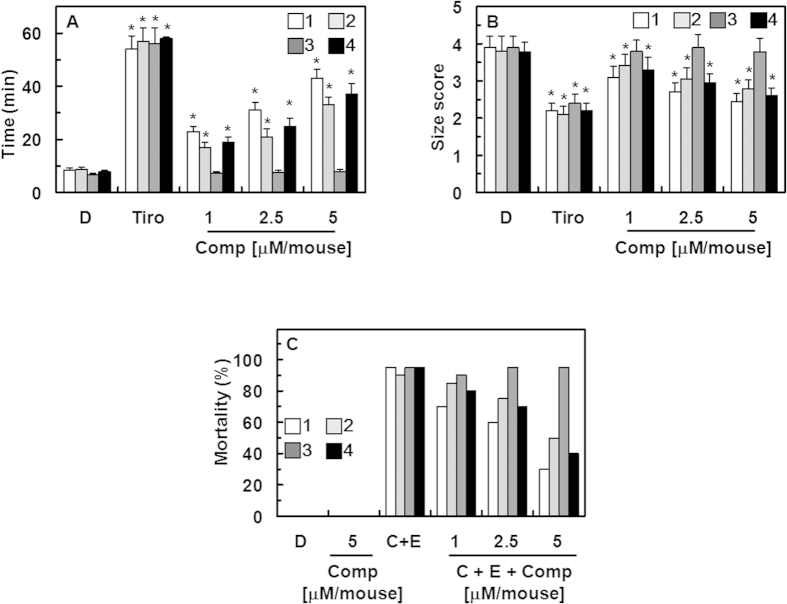
Effects of compounds 1–4 on arterial thrombosis and on acute thrombosis. (**A**) Time to large thrombus formation by compound **1** (white box), **2** (light gray box), **3** (dark gray box), and **4** (black box). Tirofiban (Tiro) was used as a positive control. (**B**) The size score of the thrombus at 60 min after FeCl_3_-treatment as described in “Materials and Methods”. (**C**) After each compound was injected intravenously, a mixture of collagen (**C**, 500 μg/kg) plus epinephrine (**E**, 50 μg/kg) was injected into the tail vein of mice to induce acute thrombosis 6 h later. Then, mice (20 mice per group) were carefully examined for 15 min to determine whether the mouse was paralyzed, dead, or recovered from the acute thrombotic challenge. D = 0.2% DMSO used as the vehicle control. *p < 0.05 vs. DMSO.

The mouse model of ferric chloride (FeCl_3_)-induced carotid artery thrombosis^25^ has been commonly used to assess antiplatelet effects. The time to thrombus formation and the size of the resulting thrombi are summarized in Fig. 4. Data showed that endothelial injury after FeCl_3_ treatment in control mice led to the growth of large thrombi at 8.5 ± 0.9 min and the antiplatelet GP IIb/IIIa inhibitor, tirofiban, significantly slowed the growth of large thrombi at 56.5 ± 5.4 min. Compounds **1**, **2**, and **4** significantly slowed the growth of thrombi (Fig. 4A). We also examined the effect of each compound on thrombus size at 60 min after FeCl_3_-induced endothelial injury (Fig. 4B). Results showed that compounds **1**, **2**, and **4** reduced FeCl_3_-induced thrombus formation. In addition, the results of *in vivo* pulmonary thrombosis model are shown in Fig. 4C. An intravenous injection of a mixture of collagen and epinephrine to mice induced massive pulmonary thrombosis causing acute paralysis, leading ultimately to sudden death (90–95% mortality). The mortalities in compounds **1-**, **2-**, and **4-**treated groups decreased significantly compared to that in collagen- and epinephrine-treated group (Fig. 4C).

### Effects of the isolated compounds on protein kinase C activation and intracellular Ca^2+^ mobilization

**Figure 5:**
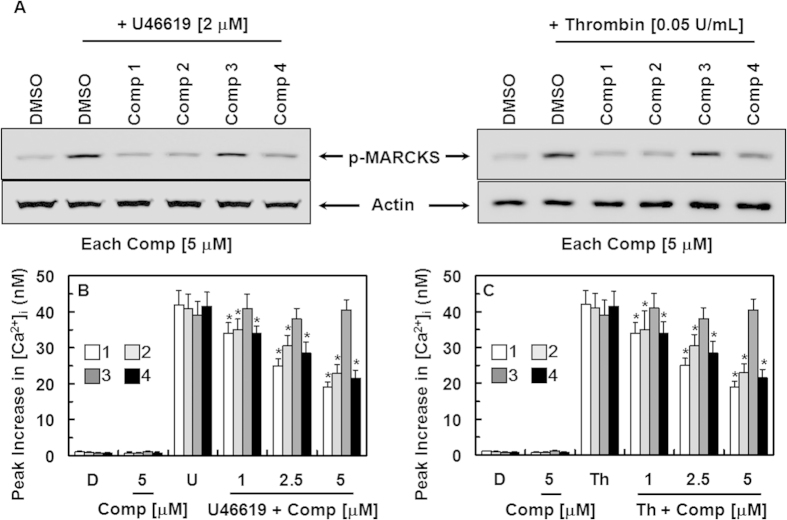
Effects of compounds 1–4 on PKC activation and intracellular calcium mobilization. (**A**) Washed human platelets were incubated with DMSO or each compound (5 μM) at 37 °C for 10 min, and were stimulated with U46619 (2 μM, left) or thrombin (0.05 U/mL, right) for another 1 min. Phospho-MARCKS in the platelet lysates was detected using western blotting (cropped images from full-length gels). (**B,C**) Fura-2-loaded human platelets were incubated with DMSO (**D**), compound **1** (white box), **2** (light gray box), **3** (dark gray box), and **4** (black box) at 37 °C for 10 min in the presence of 1 mM extracellular Ca^2+^, followed by the addition of U46619 (B, 2 μM) or thrombin (**C**, 0.05 U/mL) to trigger the increase in [Ca^2+^]_i_. The data represent the means ± SEM of three independent experiments performed in triplicate. ^*^p < 0.05 vs. U46619 (**B**) or Th (**C**) alone.

We next investigated the selectivity of each compound for the signaling pathways that regulate platelet aggregation. Upon platelet stimulation by agonists, phospholipase C (PLC) hydrolyzes phosphatidylinositol 4,5-bisphosphate to diacylglycerol and inositol-1,4,5-triphosphate, which promote the activation of protein kinase C (PKC) and increase cytosolic Ca^2+^, respectively^26^. PKC and Ca^2+^ act synergistically to induce the granular secretion and activation of glycoprotein (GP) IIb/IIIa, the receptor responsible for the final step of platelet aggregation^26^. In this study, the effect of each compound on PKC activation was determined by measuring the phosphorylation of MARCKS, which is a major substrate of PKC in human platelets^27^. At the concentrations required to prevent platelet aggregation, compounds **1**, **2**, and **4** inhibited U46619- (left) and thrombin-induced (right) MARCKS phosphorylation (Fig. 5A). The changes in intracellular [Ca^2+^]_I_ were measured in U46619- and thrombin-stimulated, fura-2-loaded platelets by monitoring the fluorescence of fura-2. Compounds **1**, **2**, and **4** inhibited the U46619- (Fig. 5B) and thrombin-induced increases (Fig. 5C) in [Ca^2+^]_i_.

### Effects of the isolated compounds on thrombin and FXa activity

**Figure 6:**
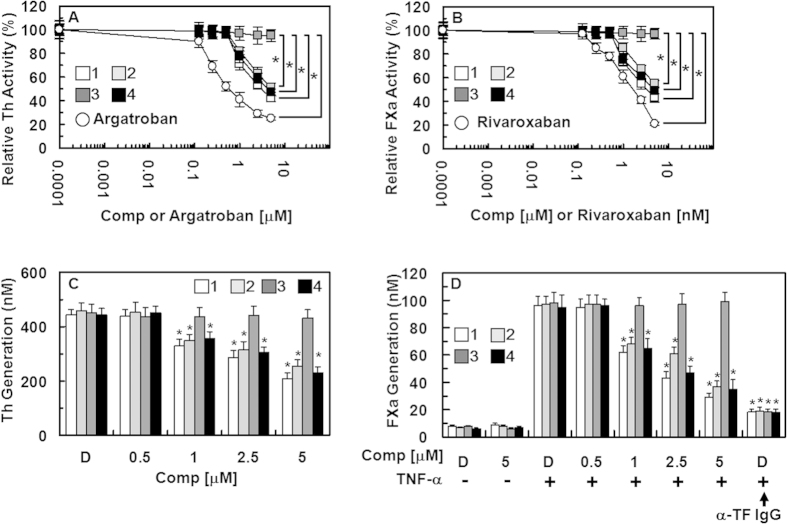
Effects of compounds 1-4 on the inactivation and production of thrombin and factor Xa. (**A**) Inhibition of thrombin (Th) by compound **1** (white box), **2** (light gray box), **3** (dark gray box), and **4** (black box) was measured using a chromogenic assay, as described in the “Materials and Methods” section. (**B**) The inhibition of factor Xa (FXa) by each compound was also monitored using a chromogenic assay, as described in the “Materials and Methods” section. Argatroban (**A**) or rivaroxaban (**B**) was used as a positive control. (**C**) The HUVEC monolayer was pre-incubated with FVa (100 pM) and FXa (1 nM) for 10 min with the indicated concentrations of each compound. Prothrombin was added at a final concentration of 1 μM and prothrombin activation was determined after 30 min, as described in the “Materials and Methods” section. (**D**) HUVECs were pre-incubated with the indicated concentrations of each compound for 10 min. TNF-α (10 ng/mL for 6 h)-stimulated HUVECs were incubated with FVIIa (10 nM) and FX (175 nM) in the absence or presence of anti-TF IgG (25 μg/mL); the FXa production was determined as described in the “Materials and Methods” section. *p < 0.05 vs. 0.0001 μM each compound, or argatroban (**A**) or 0.0001 μM each compound, or 0.0001 nM rivaroxaban (**B**), DMSO (**C**) or TNF-α alone (**D**).

To elucidate the mechanism responsible for the inhibition of coagulation by compounds **1**, **2**, and **4**, the inhibition of thrombin and FXa activity was determined using chromogenic substrates. Treatment with compounds **1**, **2**, and **4** resulted in a dose-dependent inhibition of the amidolytic activity of thrombin, indicating a direct inhibition of thrombin activity by compounds **1**, **2**, and **4** (Fig. 6A). The direct thrombin inhibitor argatroban was used as a positive control. In addition, compounds **1**, **2**, and **4** inhibited the activity of FXa (Fig. 6B). The direct FXa inhibitor, rivaroxaban, was used as a positive control. These results are consistent with those of our antithrombin assay and therefore, suggest that the antithrombotic mechanism underlying the compound **1**, **2**, and **4**’s actions involves the inhibition of fibrin polymerization or the blood coagulation pathway or both.

### Effects of the isolated compounds on thrombin and FXa production

In a previous study, Sugo *et al.* reported that endothelial cells support prothrombin activation by FXa^28^. In the current study, pre-incubation of HUVECs with FVa and FXa in the presence of CaCl_2_ before the addition of prothrombin resulted in thrombin production (Fig. 6C). In addition, treatment with compounds **1**, **2**, and **4** caused a dose-dependent inhibition of prothrombin-produced thrombin (Fig. 6C). According to the findings reported by Rao *et al.*, the endothelium provides the functional equivalent of pro-coagulant phospholipids and supports the activation of FX^29^. Furthermore, in TNF-α-stimulated HUVECs, the activation of FX by FVIIa was dependent on TF expression^30^. Thus, we investigated the effects of compounds **1**, **2**, and **4** on the activation of FX by FVIIa. HUVECs were stimulated with TNF-α to induce TF expression, causing a 13-fold increase in the rate of FX activation by FVIIa in the stimulated HUVECs (98.5 ± 4.5 nM) compared with that in the non-stimulated HUVECs (7.5 ± 0.5 nM); these effects were abrogated by anti-TF IgG (18.4 ± 2.2 nM; Fig. 6D). In addition, pre-incubation with compounds **1**, **2**, and **4** resulted in a dose-dependent inhibition of FX activation by FVIIa (Fig. 6D). Therefore, these results suggest that compounds **1**, **2**, and **4** inhibit the production of thrombin and FXa.

### Effects of the isolated compounds on the secretion of PAI-1 and t-PA

**Figure 7:**
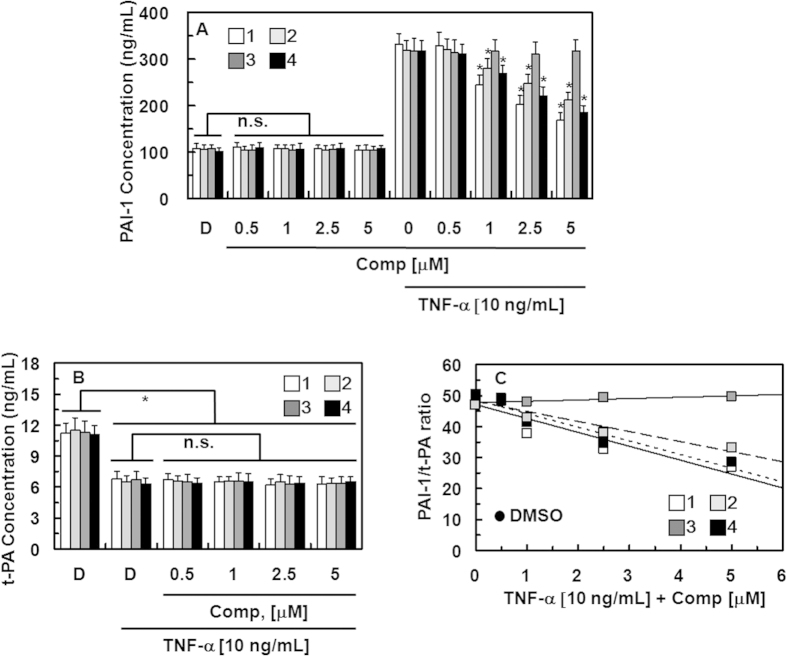
Effects of compounds 1–4 on the secretion of PAI-1 and tPA. (**A**) HUVECs were cultured with compound **1** (white box), **2** (light gray box), **3** (dark gray box), and **4** (black box) in the absence or presence of TNF-α (10 ng/mL) for 18 h and the PAI-1 concentrations in the culture media were determined as described in the “Materials and Methods” section. (**B**) HUVECs were cultured with each compound in the absence or presence of TNF-α (10 ng/mL) for 18 h and the t-PA concentrations in the culture media were determined as described in the “Materials and Methods” section. (**C**) The PAI-1/t-PA ratio in TNF-α activated HUVECs from (**A**,**B**). D = 0.2% DMSO used as the vehicle control. ^*^p < 0.05 vs. TNF-α or D alone; n.s., not significant.

TNF-α is known to inhibit fibrinolysis in HUVECs by inducing the production of PAI-I. The alteration of the balance between t-PA and PAI-1 is known to modulate coagulation and fibrinolysis^31^,^32^. To determine the direct effects of each compound on TNF-α-stimulated secretion of PAI-1, HUVECs were cultured in media with or without each compound and in the absence or presence of TNF-α for 18 h. As shown in Fig. 7A, treatment with compounds **1**, **2**, and **4** resulted in a dose-dependent inhibition of TNF-α-induced secretion of PAI-1 from HUVECs, which was significant at 1–5 μM.

TNF-α does not have a significant effect on t-PA production^33^ and the balance between plasminogen activators and their inhibitors reflects the net plasminogen-activating capacity^34^,^35^,^36^; therefore, we investigated the effect of the combination of TNF-α and compounds **1**, **2**, and **4** on the secretion of t-PA by HUVECs. Our results were consistent with those of a previous study reporting a modest decrease in the production of t-PA by TNF-α in HUVECs^37^. This decrease was not significantly altered by treatment with compounds **1**, **2**, and **4** (Fig. 7B). Collectively, these results indicate that TNF-α increased the PAI-1/t-PA ratio, which was inhibited by compounds **1**, **2**, and **4** (Fig. 7C).

## Discussion

The whole body of SSM has been used for the treatment of blood coagulation-related symptoms such as hemiplegia and is listed in the Korean Herbal Pharmacopeia and Chinese Pharmacopeia. Evidence-based researches on SSM resulted in the discovery of antithrombic peptides and proteins from the venom of SSM^5^,^6^,^7^,^8^. Despite of the several investigations on antithrombic constituents in SSM, there are few studies on the small-molecule secondary metabolites with antithrombotic and antiplatelet activities. The current study focused on the identification of small-molecule alkaloids from the whole body of SSM. Eight alkaloids including two new ones (**1** and **2**) were isolated from the EtOH extract of SSM and the structures were determined by spectroscopic analysis. Of the alkaloids isolated, a novel acylated polyamine (**1**), novel sulfated quinoline alkaloid (**2**), and indole acetic acid (**4**) were demonstrated to be the active compounds with antithrombotic and antiplatelet activities.

The PT, aPTT, fibrin polymerization, and platelet aggregation are the most established and commonly used methods to determine the efficacy of novel antithrombotic drugs^38^,^39^. In our experiments, the PT and aPTT assays were performed using human plasma to evaluate the antithrombotic effect of compounds **1**, **2**, and **4**, while fibrin polymerization and platelet aggregation were evaluated to determine the antiplatelet activity of compounds **1**, **2**, and **4**. Compounds **1**, **2**, and **4** prolonged the PT and aPTT, caused a significant decrease in the maximal rate of fibrin polymerization, and inhibited platelet aggregation without being cytotoxic to HUVECs. As shown in several studies, inflammation and coagulation are closely related processes that may have considerable effects on each other^40^,^41^. This is most apparent in platelet activation, fibrin formation, and resolution as well as in the physiological anticoagulant cascades^40^,^41^. From our data, it can be hypothesized that compounds **1**, **2**, and **4** could be promising novel anti-inflammatory mediators owing to their inhibitory effects on coagulation. Well-designed prospective studies are warranted to prove this hypothesis.

As a commercial anticoagulant, heparin has been used for the prevention of venous thromboembolic diseases for more than 60 years^42^,^43^. However, heparin has side effects such as the inability to inhibit fibrin-bound thrombin activity, ineffectiveness in congenital or acquired antithrombin deficiencies, the development of thrombocytopenia, an increased risk of thromboembolic disease if the therapeutic response is not achieved, and an increased risk of bleeding if the therapeutic range is exceeded^43^,^44^. Furthermore, the amounts of available heparin are low in bovine lung or pig intestine, from which heparin is primarily extracted^44^. Therefore, the need for discovering alternative sources of anticoagulants has increased owing to the demand for safer anticoagulant therapy. Based on the current findings, small-molecule alkaloids **1**, **2**, and **4** may provide new chemotype for the development of anticoagulants provided their therapeutic effects are established.

This study demonstrated that compounds **1**, **2**, and **4** inhibited the blood coagulation pathways by inhibiting FXa and thrombin production in HUVECs. They also inhibited the TNF-α-induced secretion of PAI-1 and platelet aggregation *in vitro* and *ex vivo*. These results add to those from previous work on this topic and may be of interest to those designing pharmacological strategies for the treatment or prevention of coagulation-related vascular diseases.

## Methods

### Cell culture

Primary human umbilical vein endothelial cells (HUVECs) were obtained from Cambrex Bio Science (Charles City, IA, USA) and were maintained as described previously^45^,^46^,^47^,^48^. Briefly, cells were cultured in EBM-2 basal media supplemented with growth supplements (Cambrex Bio Science, Charles City, IA, USA) at 37 °C under 5% CO_2_ atmosphere until confluent. All experiments were performed with HUVECs at passage 3–5.

### Animals and husbandry

Male C57BL/6 mice (6–7 weeks old, weighing 27 g) were purchased from Orient Bio Co. (Sungnam, Republic of Korea) and were used after a 12-day acclimatization period. The mice were housed at five per polycarbonate cage under a controlled temperature (20–25 °C) and humidity (40–45% relative humidity) and a 12:12 h light:dark cycle. They received a normal rodent pellet diet and water *ad libitum* during acclimatization and were treated in accordance with the Guidelines for the Care and Use of Laboratory Animals issued by Kyungpook National University, Republic of Korea (IRB No. KNU 2012-13).

### Anticoagulation assay

The aPTT and PT were determined using a Thrombotimer (Behnk Elektronik, Norderstedt, Germany) per the manufacturer’s instructions and as described previously^49^. Briefly, citrated normal human plasma (90 μL) was mixed with 10 μL of heparin or each compound and was incubated for 1 min at 37 °C. Subsequently, the aPTT assay reagent (100 μL) was added and the plasma sample was incubated for an additional 1 min at 37 °C, followed by the addition of 20 mM CaCl_2_ (100 μL). The clotting times were recorded. For the PT assays, citrated normal human plasma (90 μL) was mixed with 10 μL of each compound stock solution and was incubated for 1 min at 37 °C. The PT assay reagent (200 μL), which had been pre-incubated for 10 min at 37 °C, was subsequently added and the clotting time was recorded. The PT results were expressed in seconds and as International Normalized Ratios (INR): INR = (PT sample/PT control)^ISI,^ where ISI = international sensitivity index. The aPTT results were expressed in seconds. All experimental protocol (KNUH 2012-01-010) was approved by the Institutional Review Board of Kyungpook National University Hospitals (Daegu, Republic of Korea).

### *In vivo* bleeding time

Tail bleeding times were measured using the method described by Dejana *et al.*^49^,^50^. Briefly, C57BL/6 mice were fasted overnight prior to the experiments. One hour after the i.v. administration of each compound, the tails of the mice were transected at 2 mm from their tips. The bleeding time was defined as the time elapsed until the bleeding stopped. Bleeding times exceeding 15 min were recorded as lasting for 15 min.

### *Ex vivo* clotting time

Male C57BL/6 mice were fasted overnight and each compound in 0.5% DMSO was administered by i.v. injection. One hour after the administration, arterial blood samples (0.1 mL) were collected in 3.8% sodium citrate (1/10, v/v) for the *ex vivo* aPTT and PT determination. The clotting times were determined as described above.

### Thrombin-catalyzed fibrin polymerization

Thrombin-catalyzed polymerization was determined every 6 s for 20 min by monitoring the turbidity at 360 nm using a spectrophotometer (TECAN, Männedorf, Switzerland) at ambient temperature. Control plasma and plasma incubated with each compound were diluted three-fold in DMSO and clotted with thrombin (final concentration: 0.5 U/mL). The maximum polymerization rate (Vmax, ΔmOD/min) from each absorbance curve was recorded^51^.

### *In vitro* and *ex vivo* platelet aggregation assay

The *in vitro* platelet aggregation study was performed according to a previously reported method^52^,^53^. Washed human platelets were incubated with the indicated concentration of each compound in DMSO for 1, 3, 5, or 10 min. They were subsequently stimulated by U46619 (2 μM) in 0.9% saline solution at 37 °C for 5 min. Platelet aggregation was recorded using an aggregometer (Chronolog, Havertown, PA, USA). For the *ex vivo* aggregation assay, male mice were fasted overnight and the indicated concentration of each compound in DMSO was administered by intravenous (i.v.) injection. After 24 h, platelet-rich plasma (10^9^ platelets/mL) in a volume of 240 μL was incubated at 37 °C for 1.5 min in the aggregometer under continuous stirring at 1000 rpm and subsequently stimulated with U46619 (2 μM). Platelet aggregation was recorded as described above.

### Arterial thrombosis animal model

The FeCl_3_-induced thrombosis mouse model was established as previously described^25^. Male C57BL/6 mice were fasted overnight and were administered each indicated compound in DMSO by intravenous injection. Then, mice were anesthetized using 3% isoflurane (Forane®, Choongwae Pharma. Corp., Seoul, Korea) and injected intravenously with 0.1 mL of 0.1% rhodamine 6 G (Sigma). A testicular artery (200 μm in diameter) was carefully exposed and a cotton thread (0.2 mm in diameter) saturated with 0.25 mol/L FeCl_3_ was applied to the adventitial surface. After 5 min, the cotton thread was removed, and the wound was flushed with saline solution. Thrombus formation was monitored at 35 °C by 3-dimensional imaging as previously described^54^. The size and time of thrombus formation were monitored, and the findings were categorized as follows: score 0 indicates no thrombus; 1 indicates small thrombus (50 μm × 75 μm); 2 indicates medium-sized thrombus (100 μm × 150 μm); and 3 indicates large thrombus (200 μm × 300 μm). The time from FeCl_3_-mediated endothelial injury to occlusion of the testicular artery by a large thrombus was measured.

### Acute thrombosis induced by a combination of collagen and epinephrine in mice

Male C57BL/6 mice were fasted overnight and divided into groups of 10 animals. Each compound suspended in DMSO was administered to mice intravenously. A mixture of collagen (500 μg/kg) plus epinephrine (50 μg/kg) was injected to the tail vein of mice to induce acute thrombosis 1 h later. Each mouse was carefully examined for 15 min to determine whether the mouse was paralyzed, dead, or recovered from the acute thrombotic challenge. For statistical analysis, five separated experiments were performed.

### Western blotting

Total cell extracts were prepared by lysis of the HUVECs and the protein concentration was determined using Bradford assay. Equal amounts of protein were separated by SDS-PAGE (10%) and electroblotted overnight onto an Immobilon membrane (Millipore, Billerica, MA, USA). The membranes were blocked for 1 h with 5% low-fat milk powder in TBS (50 mM Tris-HCl, pH 7.5, 150 mM NaCl) containing 0.05% Tween^®^ 20. They were subsequently incubated with phospho-MARCKS (Santa Cruz, CA, USA) for 1.5 h at room temperature, followed by incubation with horseradish-peroxidase-conjugated secondary antibody and ECL-detection according to the manufacturer’s instructions and as previously described^46^. β-actin (1:1000, Santa Cruz, CA, USA) was used as a loading control.

### Measurement of the intracellular Ca^2+^ mobilization

The intracellular Ca^2+^ mobilization ([Ca^2+^]_i_) of platelets was measured as described previously^55^. Briefly, platelets were incubated with fura-2/AM (3 μM) at 37 °C for 30 min. After washing twice, the fura-2-loaded platelets were suspended in Ca^2+^-free Tyrode’s solution at a final concentration of 5 × 10^7^ platelets/mL. Calcium (1 mM) was added to the fura-2-loaded platelets 1 min before stimulation with platelet activators. The fluorescence (Ex 339 nm, Em 500 nm) was measured with a fluorescence spectrophotometer (TECAN, Männedorf, Switzerland). The [Ca^2+^]_i_ was calculated using the equation described by Grynkiewicz *et al.*^56^.

### Production of factor Xa on the surface of HUVECs

The TNF-α-stimulated (10 ng/mL for 6 h in serum-free medium) confluent monolayer of HUVECs (preincubated with the indicated concentrations of each compound for 10 min) in a 96-well culture plate was incubated with FVIIa (10 nM) in buffer B (buffer A [10 mM HEPES, pH 7.45, 150 mM NaCl, 4 mM KCl, and 11 mM glucose] supplemented with 5 mg/mL bovine serum albumin [BSA] and 5 mM CaCl_2_) for 5 min at 37 °C in the presence or absence of anti-TF IgG (25 μg/mL). FX (175 nM) was subsequently added to the cells in a final reaction mixture volume of 100 μL, and the cells were incubated for 15 min. The reaction was stopped by the addition of buffer A containing 10 mM EDTA and the amounts of FXa generated were measured using a chromogenic substrate. The changes in absorbance at 405 nm over 2 min were monitored using a microplate reader (Tecan Austria GmbH, Grödig, Austria). The initial color development rates were converted into FXa concentrations using a standard curve prepared with known dilutions of purified human FXa.

### Production of thrombin on the surfaces of HUVECs

Thrombin production by HUVECs was quantitated as previously described^49^,^57^. Briefly, HUVECs were pre-incubated in 300 μL containing each compound in 50 mM Tris-HCl buffer, 100 pM FVa, and 1 nM FXa for 10 min, followed by addition of prothrombin to a final concentration of 1 μM. After 10 min, duplicate samples (10 μL each) were transferred to a 96-well plate containing 40 μL of 0.5 M EDTA in TBS per well to terminate the prothrombin activation. Activated prothrombin was determined by measuring the rate of hydrolysis of S-2238 (a thrombin substrate) at 405 nm. Standard curves were prepared with known amounts of purified thrombin.

## Additional Information

**How to cite this article**: Lee, W. *et al.* Antithrombotic and antiplatelet activities of small-molecule alkaloids from *Scolopendra subspinipes mutilans. Sci. Rep.* **6**, 21956; doi: 10.1038/srep21956 (2016).

## Supplementary Material

Supplementary Information

## Figures and Tables

**Table 1:** Anticoagulant activity of compounds 1, 2, 3 and 4 from SSM^a^.

Sample	Dose	aPTT (s)	PT (s)	PT (INR)
*In vitro* coagulant assay				
Control	Saline	23.4 ± 0.2	12.4 ± 0.4	1.00
Comp 1	0.5 μM	24.8 ± 0.4	12.8 ± 0.4	1.08
	1.0 μM	33.2 ± 0.5*	15.6 ± 0.2^*^	1.73^*^
	2.5 μM	43.0 ± 0.3^*^	22.4 ± 0.4^*^	4.13^*^
	5.0 μM	57.5 ± 0.5^*^	28.5 ± 0.5^*^	7.37^*^
Comp 2	0.5 μM	24.0 ± 0.8	12.6 ± 0.2	1.04
	1.0 μM	31.6 ± 0.2^*^	16.4 ± 0.4^*^	1.96^*^
	2.5 μM	41.3 ± 0.4^*^	21.6 ± 0.6^*^	3.79^*^
	5.0 μM	52.6 ± 0.6^*^	26.2 ± 0.4^*^	6.02^*^
Comp 3	0.5 μM	24.2 ± 0.6	12.5 ± 0.3	1.02
	1.0 μM	23.8 ± 0.4	12.4 ± 0.4	1.00
	2.5 μM	23.2 ± 0.5	12.6 ± 0.6	1.04
	5.0 μM	24.4 ± 0.6	13.5 ± 0.5	1.23
Comp 4	0.5 μM	24.2 ± 0.6	12.8 ± 0.6	1.08
	1.0 μM	32.8 ± 0.4^*^	17.6 ± 0.8^*^	2.32^*^
	2.5 μM	43.7 ± 0.4^*^	23.6 ± 0.5^*^	4.69^*^
	5.0 μM	55.8 ± 0.7^*^	27.2 ± 0.7^*^	6.59^*^
Heparin	5.0 μM	60.2 ± 0.8^*^	30.4 ± 0.8^*^	8.60^*^
*In vivo* bleeding time (i.v. injection)				
Sample	Dose	Tail bleeding time (s)	n	
Control	Saline	32.2 ± 1.0	5	
Comp 1	1.73 μg/mouse	44.6 ± 1.6^*^	5	
	3.46 μg/mouse	58.2 ± 1.2^*^	5	
Comp 2	1.21 μg/mouse	40.4 ± 1.2^*^	5	
	2.41 μg/mouse	56.8 ± 1.0^*^	5	
Comp 3	0.81 μg/mouse	33.3 ± 1.2^*^	5	
	1.61 μg/mouse	35.0 ± 1.0^*^	5	
Comp 4	0.87 μg/mouse	42.2 ± 0.8^*^	5	
	1.75 μg/mouse	59.2 ± 1.2^*^	5	
Heparin	36.0 μg/mouse	71.4 ± 1.2^*^	5	

^a^Each value represents the means ± SEM (n = 5).

*p < 0.05 as compared to control.

**Table 2:** *Ex vivo* coagulation time of compounds 1–4 from SSM^a^.

Sample	Dose	aPTT (s)	PT (s)	PT (INR)
Control	Saline	30.2 ± 0.6	12.8 ± 0.4	1.00
Comp 1	1.73 μg/mouse	45.9 ± 0.8^*^	18.2 ± 0.8^*^	2.33^*^
	3.46 μg/mouse	59.5 ± 1.0^*^	24.2 ± 1.2^*^	4.61^*^
Comp 2	1.21 μg/mouse	40.4 ± 0.6^*^	15.9 ± 0.5^*^	1.68
	2.41 μg/mouse	48.8 ± 0.8^*^	20.8 ± 0.6^*^	3.21^*^
Comp 3	0.81 μg/mouse	31.2 ± 0.5	12.6 ± 0.4	0.96
	1.61 μg/mouse	30.8 ± 0.8	13.0 ± 0.9	1.04
Comp 4	0.87 μg/mouse	42.1 ± 0.7^*^	17.4 ± 0.6^*^	2.09^*^
	1.75 μg/mouse	55.3 ± 1.0^*^	22.4 ± 0.8^*^	3.83^*^

^a^Each value represents the means ± SEM (n = 5).

^*^p < 0.05 as compared to control.
