# Supplementary material for: Antithrombotic and antiplatelet activities of small-molecule alkaloids from Scolopendra subspinipes mutilans
Source: Sci Rep. 2016 Feb 24;6:21956. doi: 10.1038/srep21956 (PMC4764974; doi:10.1038/srep21956)
Supplement: Supplementary Information [file srep21956-s1.doc]

# Antithrombotic and antiplatelet activities of small molecule alkaloids from *Scolopendra subspinipes mutilans*

# Wonhwa Lee ^1,2†^, JungIn Lee^3†^, Roshan Kulkarni^3^, Mi-Ae Kim^4^, Jae Sam Hwang^4^, MinKyun Na^3^*, and Jong-Sup Bae^1^*

^1^College of Pharmacy, CMRI, Research Institute of Pharmaceutical Sciences, Kyungpook National University, Daegu 702-701, Republic of Korea; ^2^BK21 Plus KNU Biomedical Convergence Program, Department of Biochemistry and Cell Biology, School of Medicine, Kyungpook National University, Daegu 702-701, Republic of Korea; ^3^College of Pharmacy, Chungnam National University, Daejeon, 305-764, Republic of Korea; ^4^Department of Agricultural Biology, The National Academy of Agricultural Science, RDA, 166 Nongsaengmyoungro, Wanju-gun, 565-851, Republic of Korea

Running title: Anti-coagulant effects of alkaloids from *Scolopendra subspinipes mutilans*

# ^†^First two authors contributed equally to this work

# * Corresponding Authors:

# MinKyun Na, Ph.D.

College of Pharmacy, Chungnam National University

Daejeon 305-764, Republic of Korea

# Phone, 82-42-821-5925; Fax, 82-42-823-6566

# E-mail: mkna@cnu.ac.kr

# and

# Jong-Sup Bae, Ph.D.

College of Pharmacy, Research Institute of Pharmaceutical Sciences, Kyungpook National University

# 80 Daehak-ro, Buk-gu, Daegu 702-701, Republic of Korea

# Phone, 82-53-950-8570; Fax, 82-53-950-8557

# E-mail: baejs@knu.ac.kr

**Supplementary Materials and Methods**

**Reagents**

Dried individuals of SSM were purchased from an herbal market at Geumsan, Korea, and identified by one of the authors (M. Na). A voucher specimen (CNU-INS 1408) has been deposited at the Pharmacognosy Laboratory of the College of Pharmacy, Chungnam National University, Daejeon, Korea. TNF-α was purchased from Abnova (Taipei, Taiwan). The anti-tissue factor (TF) antibody was purchased from Santa Cruz Biotechnology, Inc. (Santa Cruz, CA, USA). The thromboxane A2 (TXA2) analogue U46619 was purchased from Calbiochem-Novabiochem Corp. (San Diego, CA, USA). Factor V, VII, VIIa, X, and Xa, and antithrombin III (AT III), prothrombin, and thrombin were obtained from Haematologic Technologies (Essex Junction, VT, USA). The aPTT assay reagent and PT reagents were purchased from Fisher Diagnostics (Middletown, VA, USA); the chromogenic substrates (S-2222 and S-2238) were purchased from Chromogenix AB (Mölndal, Sweden). Rivaroxaban and argatroban (a direct FXa and FIIa inhibitor, respectively) were purchased from Santa Cruz Biotechnology, Inc. (Dallas, TX, USA). The plasminogen activator inhibitor-1 (PAI-1) and tissue plasminogen activator (t-PA) enzyme-linked immunosorbent assay (ELISA) kits were purchased from American Diagnostica, Inc. (Stamford, CT, USA). All other reagents were of the highest commercially available grade.

**General Experimental Procedures**

IR data were recorded on a Thermo Electron US/Nicolet380 (Madison, WI, USA). NMR experiments were carried out using a Bruker Avance III (600 MHz) spectrometer. HRESIMS data were obtained on a JMS 700 high-resolution mass spectrometer (JEOL, Tokyo, Japan). Vacuum-liquid chromatography (VLC) was conducted on Merck silica gel (70−230 mesh), and medium-pressure liquid chromatography (MPLC) was carried out utilizing a Biotage Isolera apparatus equipped with a reversed-phase C18 SNAP Cartridge KPC18- HS (340 g, Biotage AB, Uppsala, Sweden). Preparative reversed phase HPLC (prep HPLC) separation was carried out on a YMC C_18_ column (250 × 20.0 mm, 5 μm) or on a Kinetex Biphenyl column (250 × 21.5 mm, 5 μm) at 5 mL/min flow rate.

**Extraction and Isolation**

The dried SSM (521 g) were extracted with ethanol (5 L × 4) at room temperature and the extract obtained was concentrated under vacuum to yield a brownish ethanol extract (110.0 g). The extract was suspended in water and fractionated successively using ethyl acetate (EtOAc) and *n*-butanol (BuOH) to yield EtOAc-soluble fraction (SS-1, 60.0 g), BuOH-soluble fraction (SS-2, 8.0 g), and residue (SS-3, 40.0 g). The EtOAc-soluble fraction (SS-1) was subjected to VLC using hexane/EtOAc 40:1, 20:1, 10:1, and 4:1; hexane/EtOAc/MeOH 2/1/0.2; CHCl_3_/MeOH 6:1; CHCl_3_/MeOH/H_2_O 3:1:0.1 followed by washing with MeOH as eluents to yield 8 fractions (SS-1a-h). Fraction SS-1d (8.0 g) eluting with hexane/EtOAc 4:1 was partitioned with hexane and MeOH to yield jineol (60.0 mg). Fraction SS-1f (1.6 g) eluting with CHCl_3_/MeOH 6:1 was subjected to prep HPLC using a gradient of MeOH in water from 10 to 100% to isolate 3-methoxy-8-hydroxy-quinolin-2-one (*t*_R_, 42.0 min, 22.0 mg) and 3,4-dimethoxy-8-hydroxy-quinolin-2-one (*t*_R_, 52.0 min, 36.0 mg). Fraction SS-1g (700 mg) eluting with CHCl_3_/MeOH/H_2_O 3:1:0.1 was separated further using prep HPLC on the Kinetex Biphenyl column using a gradient of MeOH in water from 15 to 85 % to isolate 8-hydroxy-4-quinolone (*t*_R_, 37.0 min, 3.6 mg). The BuOH-soluble fraction (SS-2, 7.4 g) was separated by VLC using hexane/EtOAc/MeOH 2/1/0.2; CHCl_3_/MeOH 8:1, 6:1, and 4:1; CHCl_3_/MeOH/H_2_O 2:1:0.1 followed by MeOH 100% as mobile phases to give six fractions (SS-2a-f). Combined fractions SS-2d and SS-2e (eluting with CHCl_3_/MeOH 6:1 and 4:1, 7.5 g) were subjected to MPLC to yield eight fractions (SS-2e-I to SS-2e-VIII). Fraction SS-2e-III (213 mg) was further separated by prep HPLC using an isocratic solvent condition of 30% MeOH. The HPLC fraction eluting between 10 to 12.5 min was combined and purified using prep HPLC with a mobile phase of 10% MeOH in water to isolate compound **2** (*t*_R_, 32.0 min, 15.0 mg). Fraction SS-2e-V (89 mg) was applied to prep HPLC using a gradient of MeOH-H_2_O (20% methanol, 25 min; 20 to 25%, 30 min; and 25 to 55%, 10 min) to isolate compound **1** (*t*_R_, 41.0 min, 1.0 mg). Fraction SS-2e-VI (85 mg) was purified by prep HPLC using an isocratic solvent of MeOH/H_2_O (1:1) to isolate scolopendrine (*t*_R_, 12.4 min, 10.0 mg).

*Compound (****1****):* yellow amorphous powder; UV (MeOH) λ_max_ (log *ε*) 240 sh (4.22), 308 (3.61) nm; IR (KBr) *ν*_max_ 3357, 1657, 1547, cm^−1^; ^1^H and ^13^C NMR, see Table 1; HR-ESI-MS m/z 369.0841 [M + Na]^+^ (calcd for C_12_H_18_N_4_O_6_SNa, 369.0845).

*Compound (****2****):* yellow amorphous powder; UV (MeOH) λ_max_ (log *ε*) 242 (4.49), 326 (3.67) nm; IR (KBr) *ν*_max_ 3385, 1606, 1550, cm^−1^; ^1^H and ^13^C NMR: see Table 1; HR-ESI-MS [M + Na]^+^ *m*/*z* 263.9935 (calcd for C_9_H_7_NO_5_S Na, 263.9943).

**Isolation of human plasma and platelets**

Human blood samples were collected in the morning from 10 healthy, fasted volunteers (age: 24-28 years, four males and six females) without cardiovascular disorders, allergy and lipid or carbohydrate metabolism disorders, and who did not received drug treatment. All subjects gave written informed consent before participation in the study. The subjects did not use addictive substances or antioxidant food supplementation, and their diet was balanced (meat and vegetables). Blood was collected in sodium citrate (0.32% final concentration, 10.9 mM) and immediately centrifuged (1,300 g × 15 min) in order to obtain plasma and pooled plasma was used for further study. Human platelets were prepared as described previously ^1, 2^. Briefly, platelet rich plasma (PRP) was prepared by centrifugation at room temperature for 15 min at 150 g. PRP was adjusted to a concentration of 1 x 10^9^ platelets/mL with use of a hemocytometer for cell counts. PRP was washed once with HEPES buffer (5 mM HEPES, 136 mM NaCl, 2.7 mM KCl, 0.42 mM NaH_2_PO_4_, 2 mM MgCl_2_, 5.6 mM glucose, 0.1% BSA (w/v), pH to 7.45) in the presence of 1mM CaCl_2_. The platelets were left at room temperature for 30 min. Ten-milliliter blood samples were used for each clotting time point measurement. The study protocol (KNUH 2012-01-010) was approved by the Institutional Review Board of Kyungpook National University Hospitals (Daegu, Republic of Korea).

**Cell viability assay**

The MTT assay was used as an indicator of cell viability. Cells were grown in 96-well plates at a density of 5 × 10^3^ cells/well. After 24 h, the cells were washed with fresh medium, followed by treatment with each compound. After a 48-h incubation period, the cells were washed and 100 μL of 1 mg/mL MTT were added to the cells, followed by incubation for 4 h. Finally, 150 μL dimethyl sulfoxide (DMSO) were added to solubilize the formazan salt formed, the amount of which was determined by measuring the absorbance at 540 nm using a microplate reader (Tecan Austria GmbH, Grödig, Austria).

**Thrombin activity assay**

Each compound in 50 mM Tris-HCl buffer (pH 7.4) containing 7.5 mM EDTA and 150 mM NaCl was mixed. Following a 2-min incubation at 37°C, thrombin solution (150 μL, 10 U/mL) was added, followed by incubation at 37°C for 1 min. S-2238 (a thrombin substrate. 150 μL, 1.5 mM) solution was subsequently added and the absorbance at 405 nm was monitored for 120 s using a spectrophotometer (TECAN, Männedorf, Switzerland).

**Factor Xa activity assay**

The FXa assay was performed using the same method as described for the thrombin activity assay, except for the use of FXa (1 U/ mL) and S-2222 as substrates instead.

**PAI-1 and t-PA ELISA**

The concentrations of PAI-1 and t-PA in HUVEC cultured supernatants were determined using ELISA kits (American Diagnostica, Inc., Stamford, CT, USA).

**Statistical Analysis**

The results were expressed as the means ± standard error of the mean (SEM) of at least three independent experiments performed in duplicate. *P* < 0.05 was considered statistically significant and was determined using the SPSS software (version 14.0, SPSS Science, Chicago, IL, USA). Statistical relevance was determined by one-way analysis of variance (ANOVA) and Tukey’s post-test.

**Full length gels for figure 5A and 5B.**

**
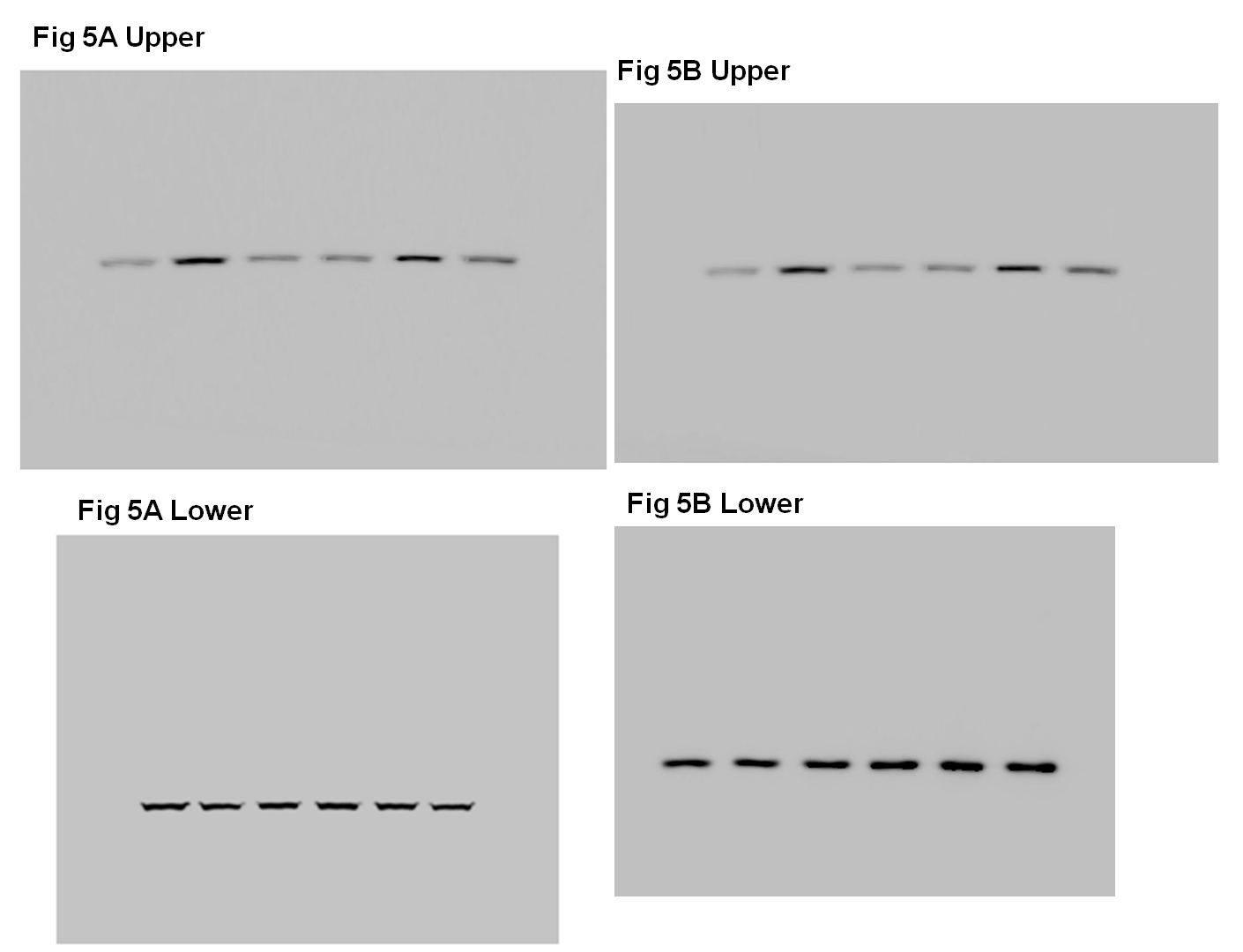
**

**Supplementary Table 1. ^1^H and ^13^C NMR Spectroscopic Data for Compound 1 in Methanol-*d*_4_**

| **1** | | | | |
| --- | --- | --- | --- | --- |
| Position |  | ^13^C, type |  | ^1^H (*J* in Hz) |
| 1 |  | 116.7 , C |  | - |
| 2 |  | 121.7, CH |  | 7.64 d (2.7) |
| 3 |  | 145.7 , C |  | - |
| 4 |  | 128.9, CH |  | 7.31 dd (2.7, 8.9) |
| 5 |  | 119.0, CH |  | 6.85 d (8.9) |
| 6 |  | 158.9, C |  | - |
| 7 |  | 170.8, C |  | - |
| 1′ |  | 39.7, CH_2_ |  | 3.44 t (7.0) |
| 2′ |  | 27.6, CH_2_ |  | 1.68 m |
| 3′ |  | 27.1, CH_2_ |  | 1.68 m |
| 4′ |  | 42.1, CH_2_ |  | 3.23 t (7.0) |
| 5′ |  | 158.6, C |  | - |

**Supplementary Table 2. ^1^H and ^13^C NMR Spectroscopic Data for Compounds 2 and 3 in Methanol-*d*_4_**

|  |  | **2** | |  | **3** | |
| --- | --- | --- | --- | --- | --- | --- |
| Position |  | ^13^C, type | ^1^H (*J* in Hz) |  | ^13^C, type | ^1^H (*J* in Hz) |
| 1 |  | - | - |  | - | - |
| 2 |  | 144.6, CH | 8.54 d (2.6) |  | 140.6, CH | 7.93 d (7.1) |
| 3 |  | 153.8, C | - |  | 109.7, CH | 6.35 d (7.1) |
| 4 |  | 117.9, CH | 7.50 d (2.6) |  | 181.0, C | - |
| 4a |  | 132.6, C | - |  | 127.9, C | - |
| 5 |  | 123.9, CH | 7.53 dd (0.7, 8.3) |  | 116.1, CH | 7.70 d (7.7) |
| 6 |  | 128.1, CH | 7.45 t (8.0) |  | 125.4, CH | 7.24 t (8.0) |
| 7 |  | 117.4, CH | 7.69 dd (0.7, 7.6) |  | 115.5, CH | 7.10 d (8.3) |
| 8 |  | 149.0, C | - |  | 148.5, C | - |
| 8a |  | 136.4, C | - |  | 132.4, C | - |

**Supplementary Table 3. Anticoagulant activity of other compounds.**

| *In vitro* coagulant assay | | | | |
| --- | --- | --- | --- | --- |
| Sample | Dose | aPTT (s) | PT (s) | PT (INR) |
| Control | saline | 23.4 ± 0.2 | 12.4 ± 0.4 | 1.00 |
| Jineol | 2.5 μM | 23.2 ± 0.4 | 12.7 ± 0.4 | 1.06 |
|  | 5.0 μM | 23.6 ± 0.6 | 12.6 ± 0.2 | 1.04 |
| 3-methoxy-8-hydroxy-quinolin-2-one | 2.5 μM | 23.3 ± 0.4 | 12.7 ± 0.5 | 1.06 |
|  | 5.0 μM | 24.0 ± 0.2 | 12.0 ± 0.3 | 0.92 |
| 3,4-dimethoxy-8-hydroxy-quinolin-2-one | 2.5 μM | 23.5 ± 0.4 | 12.2 ± 0.4 | 0.96 |
|  | 5.0 μM | 23.6 ± 0.6 | 13.4 ± 0.4 | 1.20 |
| Scolopendrine | 2.5 μM | 22.5 ± 0.8 | 13.2 ± 0.4 | 1.16 |
|  | 5.0 μM | 23.6 ± 0.4 | 12.6 ± 0.6 | 1.04 |
| Heparin | 5.0 μM | 60.2 ± 0.8^*^ | 30.4 ± 0.8^*^ | 8.60^*^ |

^a^Each value represents the means±SEM (n=5).

* p < 0.05 as compared to control.

**Supplementary Table 4. *Ex vivo* coagulation time of other compounds.**

| Sample | Dose | aPTT (s) | PT (s) | PT (INR) |
| --- | --- | --- | --- | --- |
| Control | saline | 30.2 ± 0.6 | 12.8 ± 0.4 | 1.00 |
| Jineol | 0.81 μg/mouse | 31.2 ± 0.4 | 12.3 ± 0.6 | 0.91 |
|  | 1.61 μg/mouse | 32.1 ± 1.2 | 12.4 ± 1. | 0.93 |
| 3-methoxy-8-hydroxy-quinolin-2-one | 0.96 μg/mouse | 30.9 ± 0.8 | 12.9 ± 0.6 | 1.02 |
|  | 1.91 μg/mouse | 31.2 ± 0.9 | 12.8 ± 0.7 | 1.00 |
| 3,4-dimethoxy-8-hydroxy-quinolin-2-one | 1.11 μg/mouse | 32.2 ± 0.8 | 13.1 ± 0.8 | 1.06 |
|  | 2.21 μg/mouse | 30.9 ± 0.6 | 13.2 ± 1.2 | 1.08 |
| Scolopendrine | 2.03 μg/mouse | 31.1 ± 1.2 | 12.9 ± 0.8 | 1.02 |
|  | 4.06 μg/mouse | 321 ± 1.1 | 12.7 ± 0.6 | 0.98 |

^a^ Each value represents the means±SEM (n=5).

* p < 0.05 as compared to control.

| Figures |  |
| --- | --- |
| **Figure S1** | Compounds isolated from *Scolependra subspinipes* |
| **Figure S2** | **Key HMBC ( ) and COSY ( ) correlations of compounds 1 and 2.** |
| **Figure S3** | HRESIMS spectrum of compound **1** |
| **Figure S4** | ^1^H spectrum of compound **1** |
| **Figure S5** | ^13^C spectrum of compound **1** |
| **Figure S5** | HMBC correlations of compound **1** |
| **Figure S7** | HMBC correlations of compound **1** |
| **Figure S8** | COSY correlations of compound **1** |
| **Figure S9** | ^1^H spectrum of synthetic gentisic acid |
| **Figure S10** | ^13^C spectrum of synthetic gentisic acid |
| **Figure S11** | ^1^H spectrum of synthetic agmatine sulfate |
| **Figure S12** | ^13^C spectrum of synthetic agmatine sulfate |
| **Figure S13** | HRESIMS spectrum of compound **2** |
| **Figure S14** | ^1^H spectrum of compound **2** |
| **Figure S15** | ^13^C spectrum of compound **2** |
| **Figure S16** | HMBC correlations of compound **2** |
| **Figure S17** | COSY correlations of compound **2** |
| **Figure S18** | ^1^H spectrum of compound **3** |
| **Figure S19** | ^13^C spectrum of compound **3** |
| **Figure S20** | 2D NMR correlation of compound **3** |
| **Figure S21** | ^1^H spectrum of compound **4** |
| **Figure S22** | DEPT spectrum of compound **4** |
| **Figure S23** | 2D NMR correlation of compound **4** |
| **Figure S24** | ^1^H spectrum of Jineol |
| **Figure S25** | ^13^C spectrum of Jineol |
| **Figure S26** | ^1^H spectrum of 3-methoxy-8-hydroxy-quinolin-2-one |
| **Figure S27** | ^13^C spectrum of 3-methoxy-8-hydroxy-quinolin-2-one |
| **Figure S28** | ^1^H spectrum of 3,4-dimethoxy-8-hydroxy-quinolin-2-one |
| **Figure S29** | ^13^C spectrum of 3,4-dimethoxy-8-hydroxy-quinolin-2-one |
| **Figure S30** | ^1^H spectrum of Scolopendrine |
| **Figure S31** | ^13^C spectrum of Scolopendrine |
| **Figure S32** | The effects of compounds 1-4 on reptilase-catalyzed polymerization |

**Figure S1. Compounds isolated from *Scolependra subspinipes mutilans*.**

**
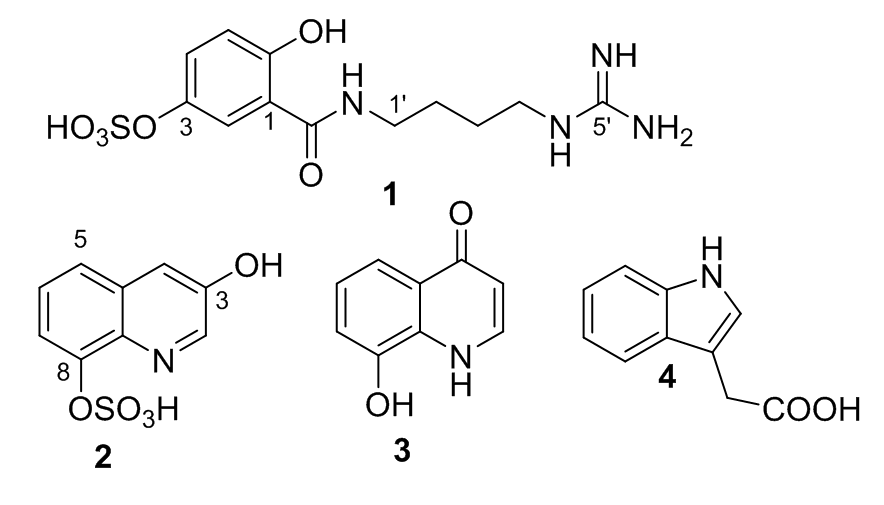
**

**Figure 2. Key HMBC ( ) and COSY ( ) correlations of compounds 1 and 2.**

**
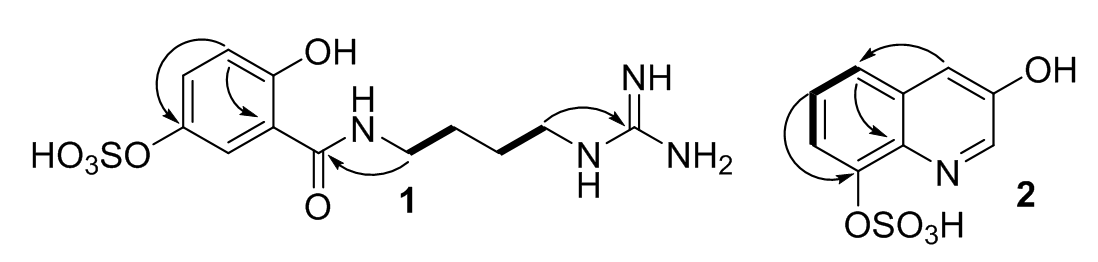
**

**Figure S3.** HRESIMS spectrum of compound **1**

**Figure S4.** ^1^H NMR of compound **1**

**Figure S4.** ^1^H NMR of compound **1** (zoomed)

**Figure S5.** ^13^C NMR of compound

**1**

**Figure S6.** HMBC correlations of compound **1**


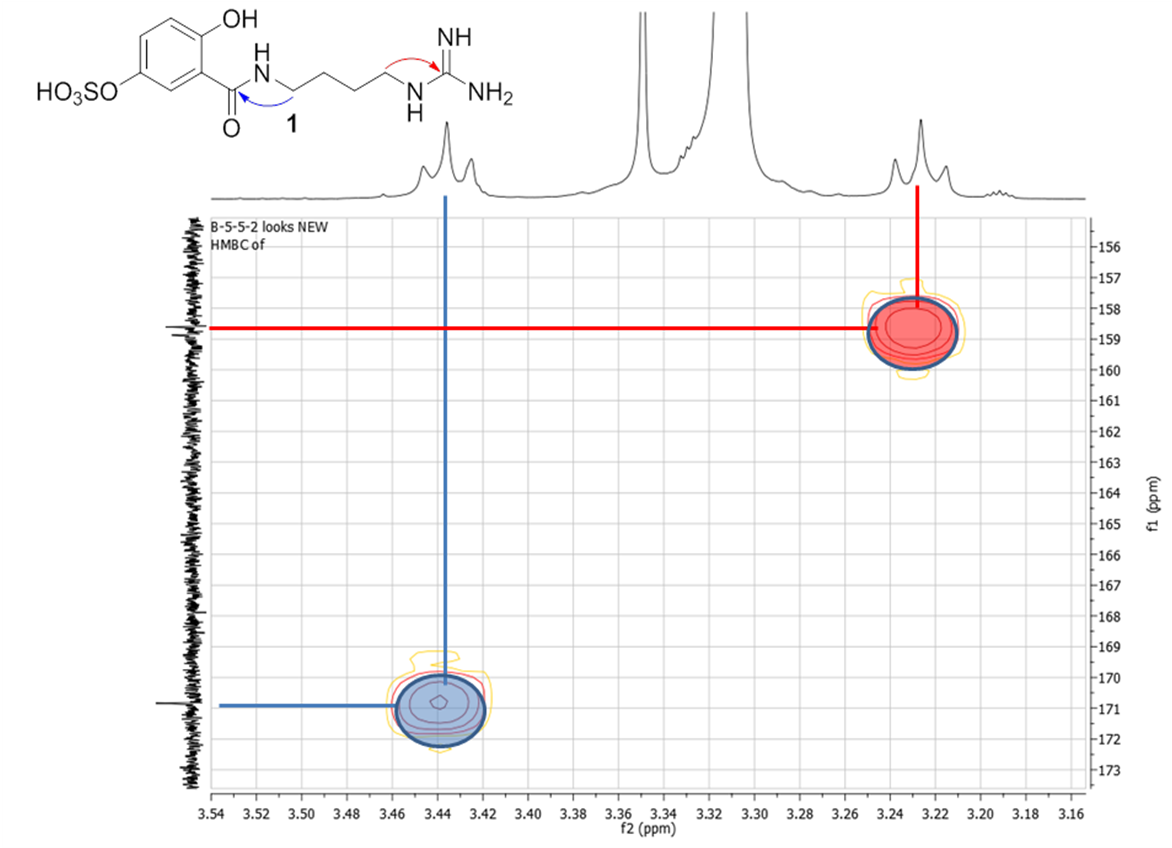


**Figure S7.** HMBC correlations of compound

**1**
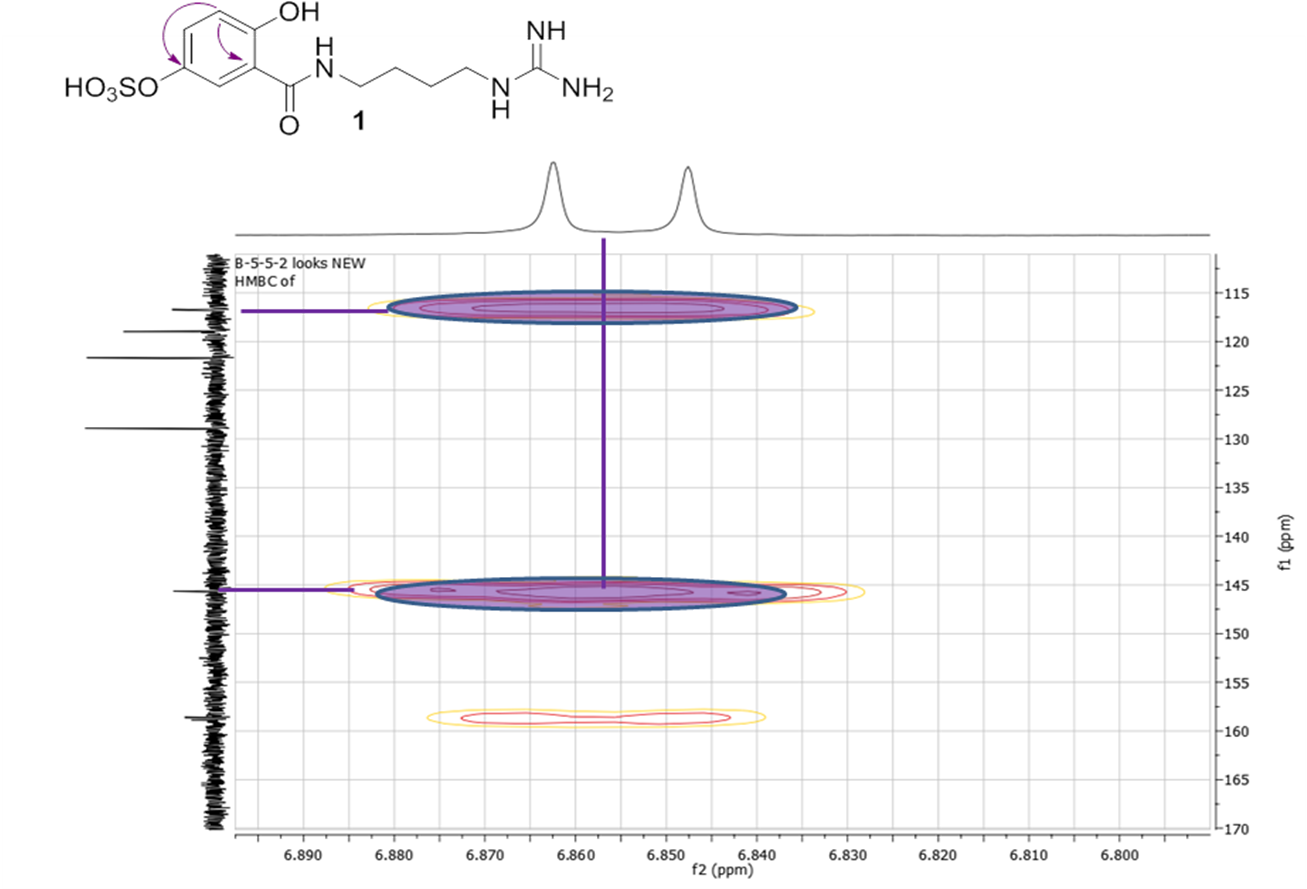


**Figure S8.** COSY correlations of compound **1**


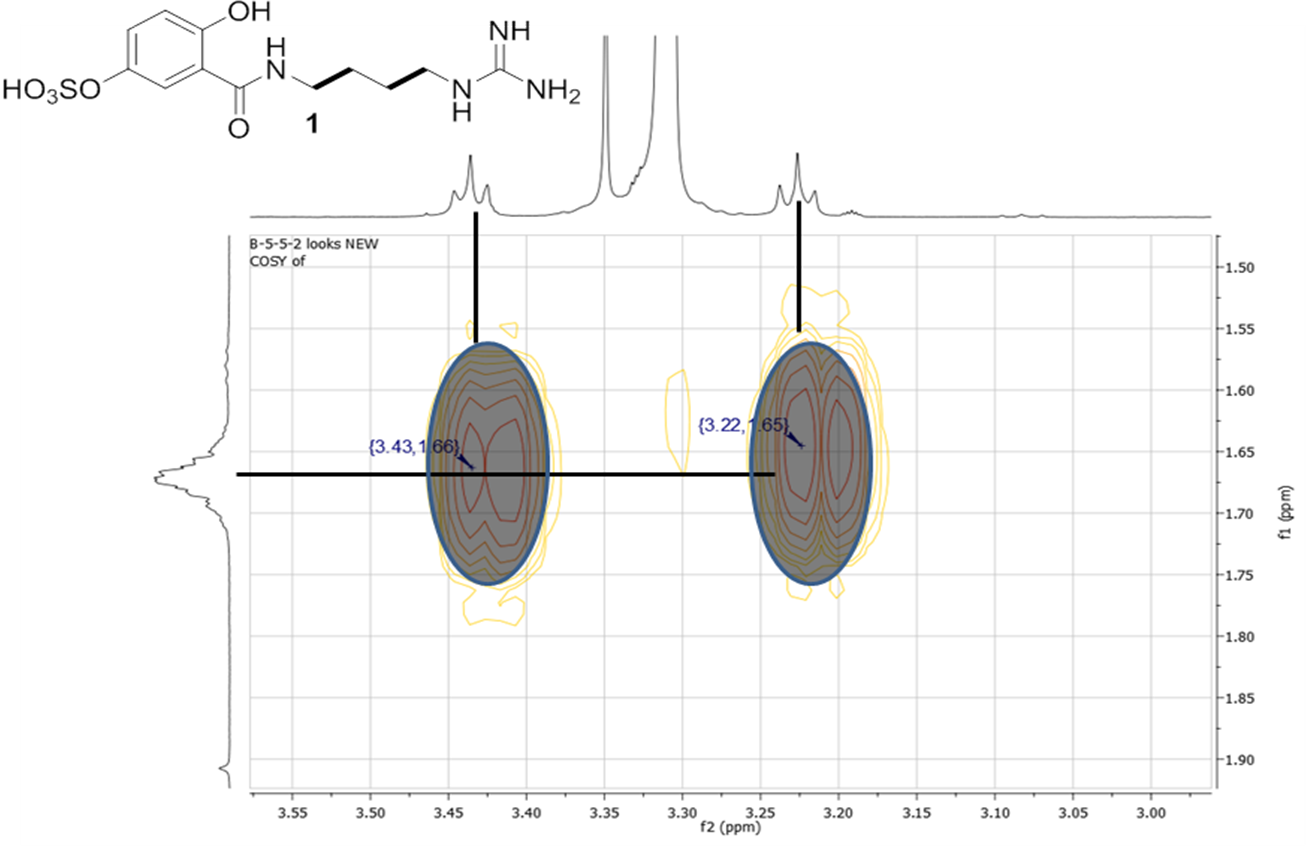


**Figure S9.** ^1^H NMR of synthetic gentisic acid

**Figure S10.** ^13^C NMR of synthetic gentisic acid

**Figure S11.** ^1^H NMR of synthetic agmatine sulfate

**Figure S12.** ^13^C NMR of synthetic agmatine sulfate

**Figure S13.**HRESIMS spectrum of compound **2**


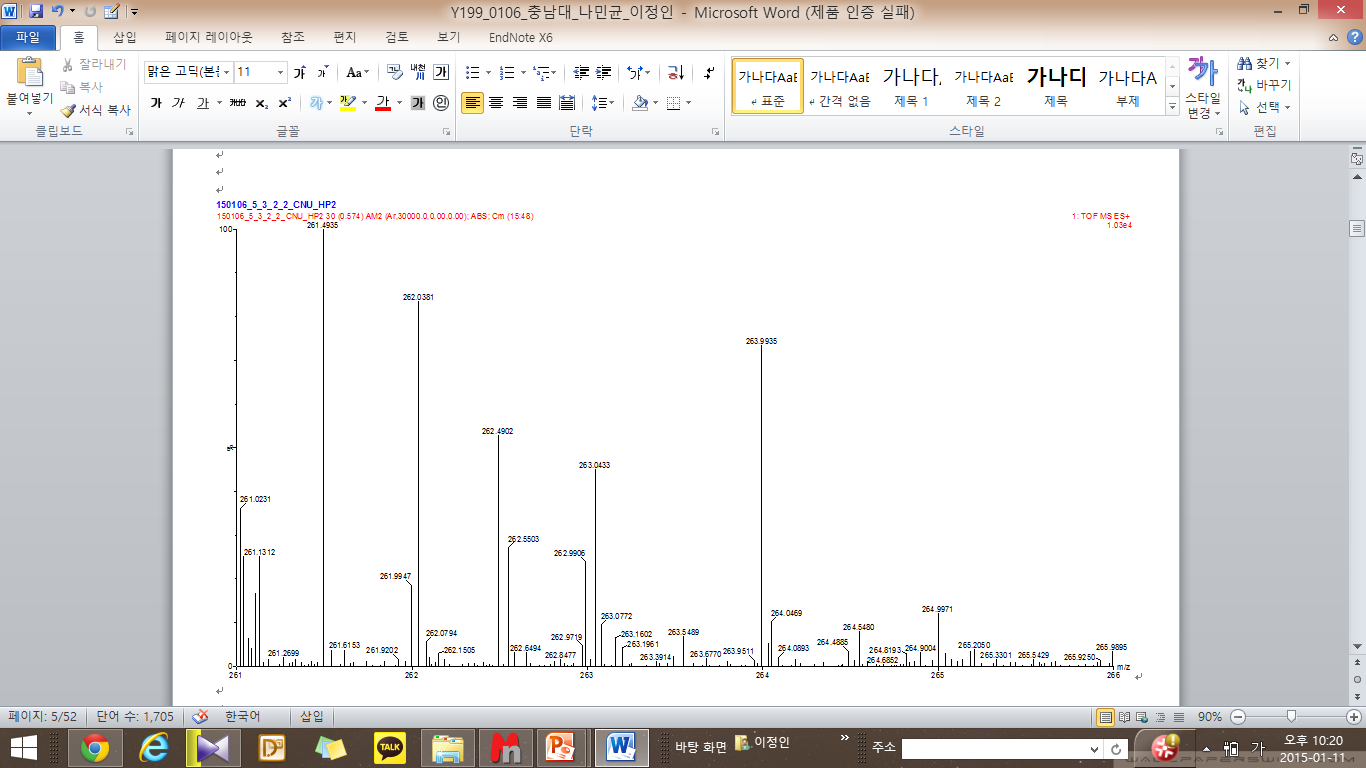


**Figure S14.**^1^H NMR of compound **2**

**Figure S15.**^13^C NMR of compound **2**

**Figure S16.**HMBC correlations of compound **2**


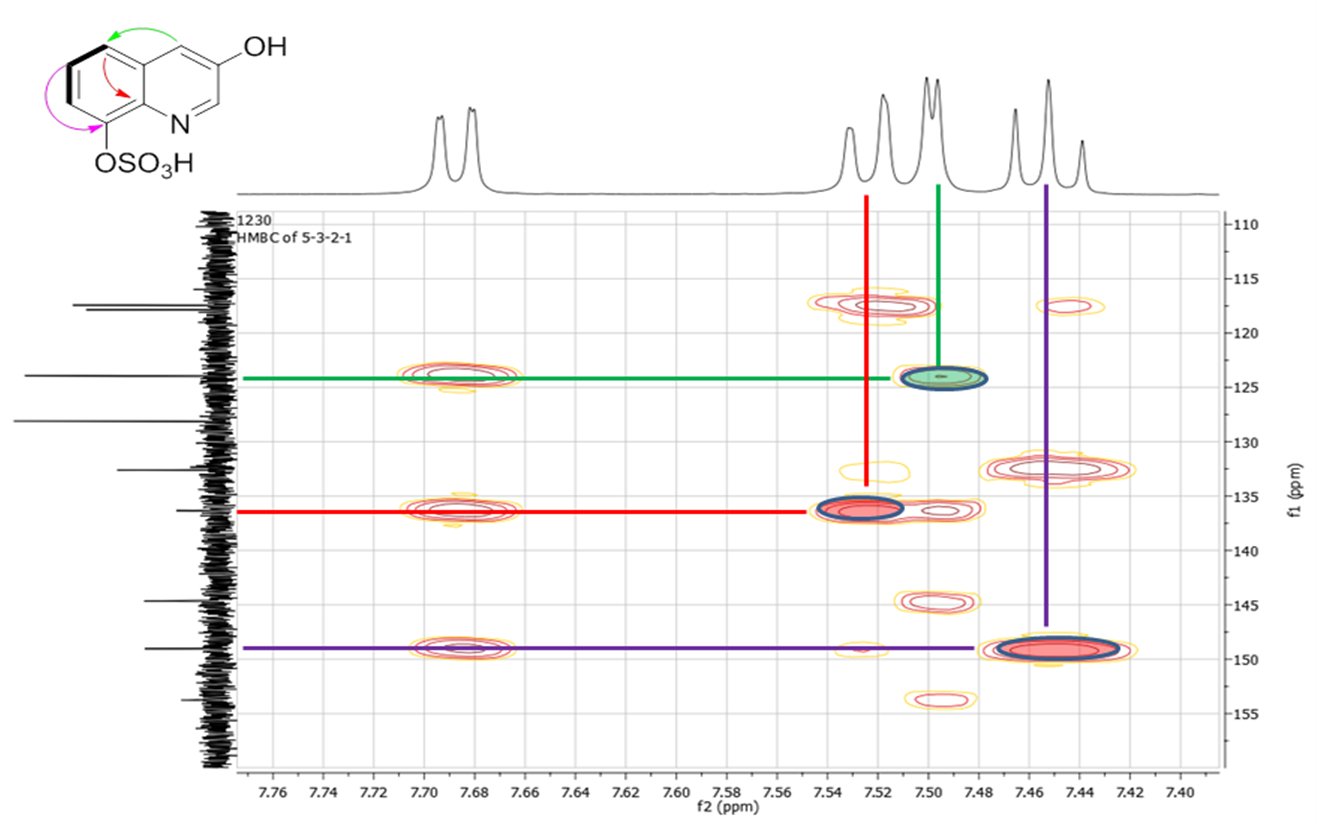


**Figure S17.** COSY correlations of compound

**2**
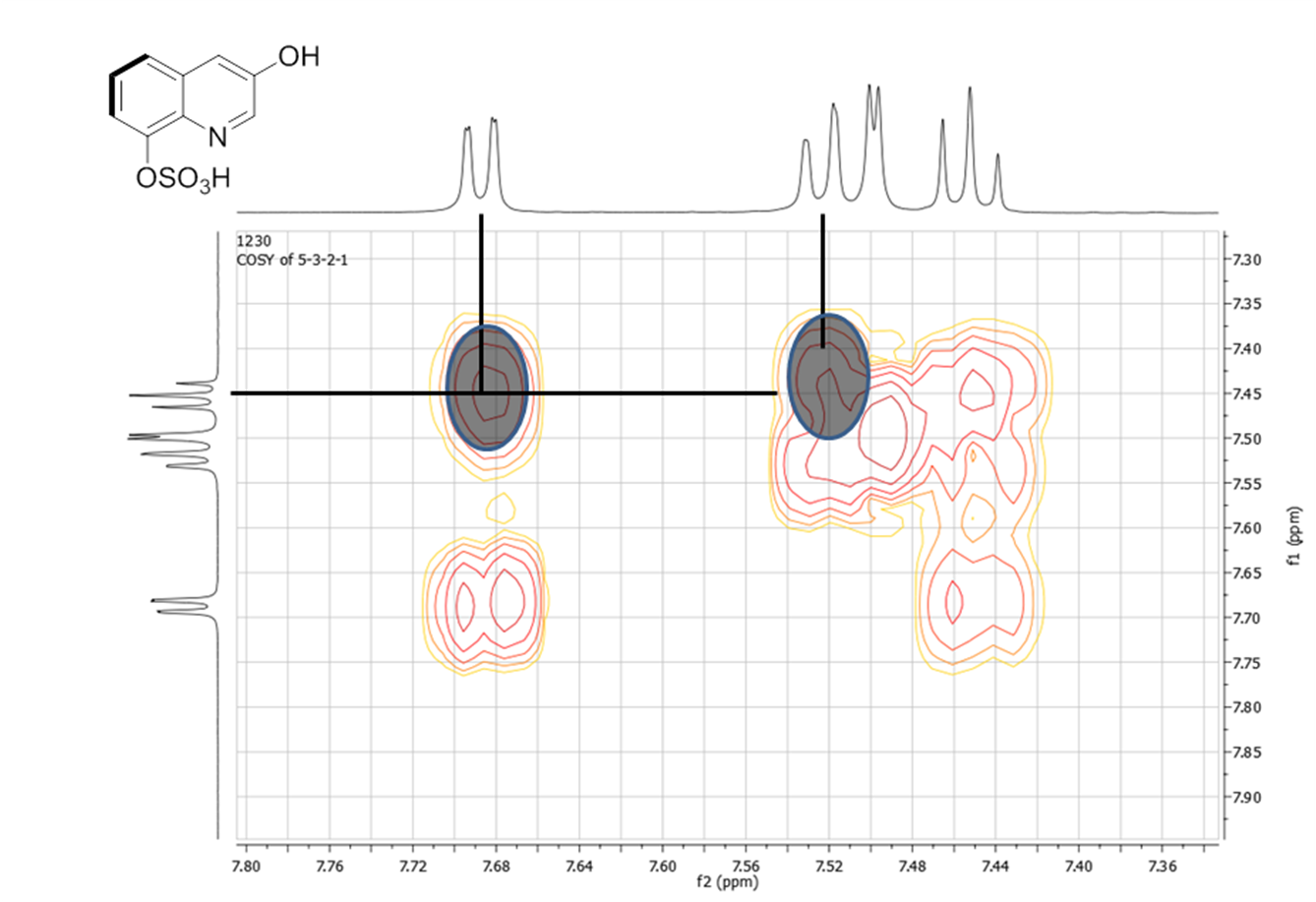


**Figure S18.** ^1^H NMR of compound **3**

**Figure S19.** ^13^C NMR of compound **3**

**Figure S20.** HMBC correlations of compound **3**


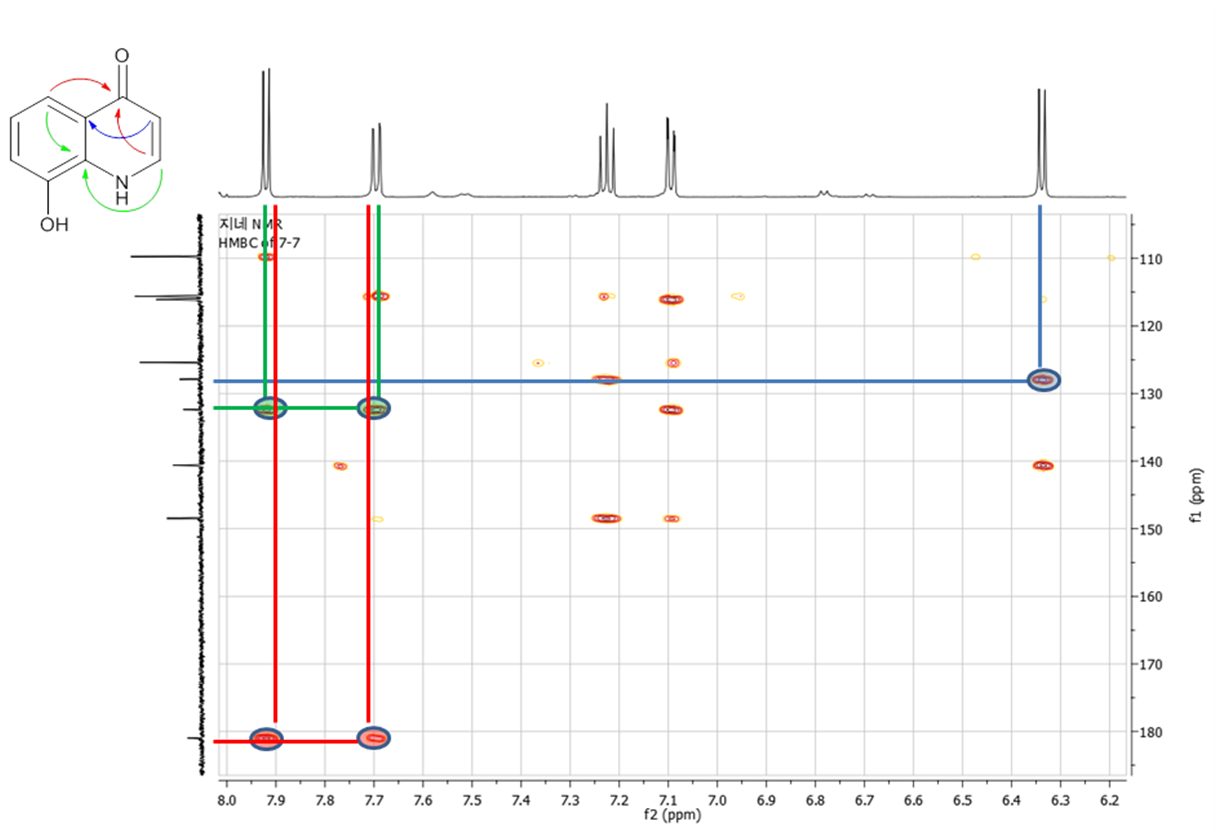


**Figure S21.** ^1^H NMR of compound **4**

**4**

**Figure S22.** DEPT NMR of compound **4**

**4**

**Figure S23.** HMBC correlations of compound **4**

**4
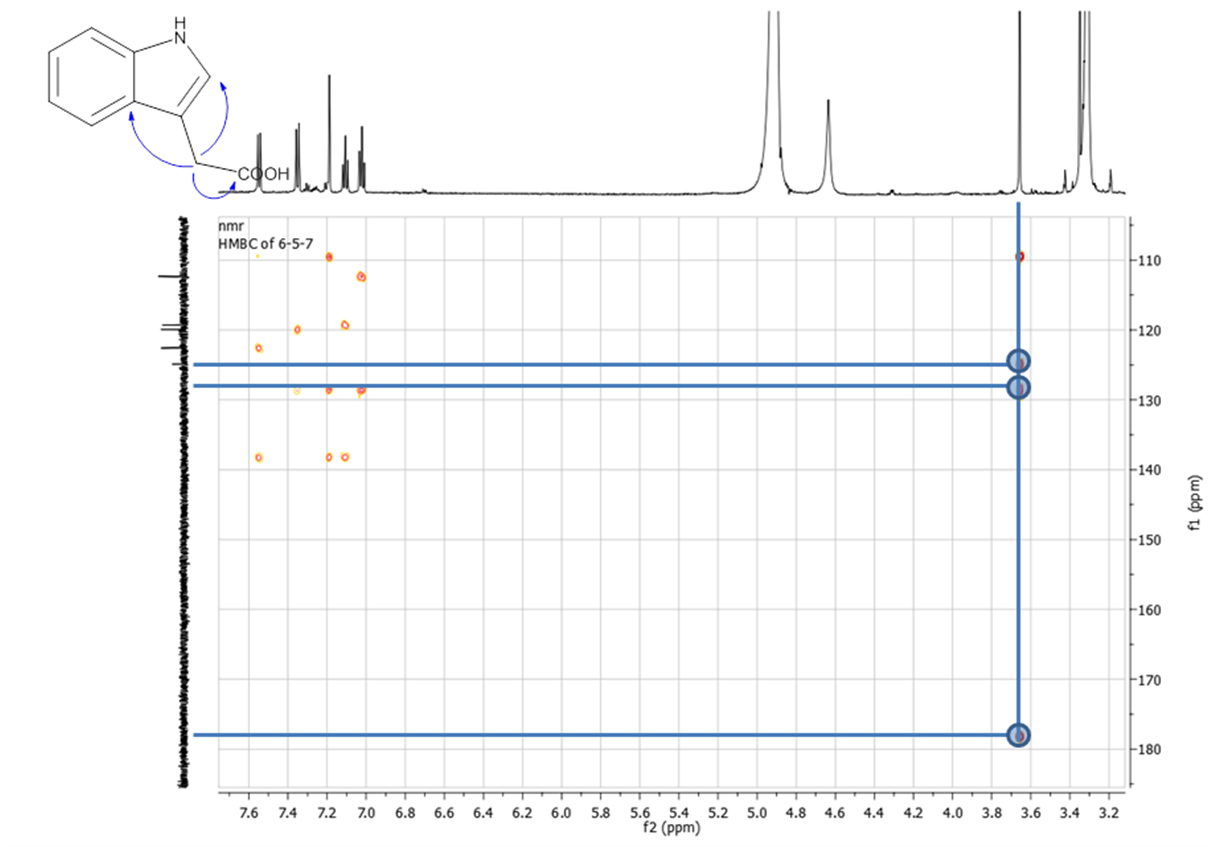
**

**Figure S24.** ^1^H NMR of Jineol

**Figure S25.** ^13^C NMR of Jineol

**Figure S26.** ^1^H NMR of 3-methoxy-8-hydroxy-quinolin-2-one

**Figure S27.** ^13^C NMR of 3-methoxy-8-hydroxy-quinolin-2-one

**Figure S28.** ^1^H NMR of 3,4-dimethoxy-8-hydroxy-quinolin-2-one

**Figure S29.** ^13^C NMR of 3,4-dimethoxy-8-hydroxy-quinolin-2-one

**Figure S30.** ^1^H NMR of Scolopendrine

**Figure S31.** ^13^C NMR of Scolopendrine

**Figure S32.** The effects of compounds 1-4 on reptilase-catalyzed polymerization


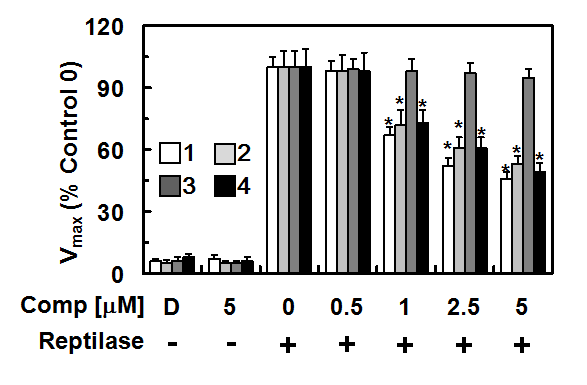


1. Franke B, Akkerman JW, Bos JL. Rapid Ca2+-mediated activation of Rap1 in human platelets. *EMBO J* 1997, **16**(2)**:** 252-259.

2. Lee W, Ku SK, Bae JS. Antiplatelet, anticoagulant, and profibrinolytic activities of baicalin. *Arch Pharm Res* 2015, **38**(5)**:** 893-903.
